# High-accuracy morphological identification of bone marrow cells using deep learning-based Morphogo system

**DOI:** 10.1038/s41598-023-40424-x

**Published:** 2023-08-17

**Authors:** Zhanwu Lv, Xinyi Cao, Xinyi Jin, Shuangqing Xu, Huangling Deng

**Affiliations:** 1 Bone Marrow Chamber, Guangzhou Kingmed Diagnostic Laboratory Group Co., Ltd., Guangzhou, 510330 China; 2 Division of Medical Technology Development, Hangzhou Zhiwei Information Technology Co., Ltd., Hangzhou, 310000 China

**Keywords:** Cancer, Diseases, Engineering

## Abstract

Accurate identification and classification of bone marrow (BM) nucleated cell morphology are crucial for the diagnosis of hematological diseases. However, the subjective and time-consuming nature of manual identification by pathologists hinders prompt diagnosis and patient treatment. To address this issue, we developed Morphogo, a convolutional neural network-based system for morphological examination. Morphogo was trained using a vast dataset of over 2.8 million BM nucleated cell images. Its performance was evaluated using 508 BM cases that were categorized into five groups based on the degree of morphological abnormalities, comprising a total of 385,207 BM nucleated cells. The results demonstrated Morphogo’s ability to identify over 25 different types of BM nucleated cells, achieving a sensitivity of 80.95%, specificity of 99.48%, positive predictive value of 76.49%, negative predictive value of 99.44%, and an overall accuracy of 99.01%. In most groups, Morphogo cell analysis and Pathologists' proofreading showed high intragroup correlation coefficients for granulocytes, erythrocytes, lymphocytes, monocytes, and plasma cells. These findings further validate the practical applicability of the Morphogo system in clinical practice and emphasize its value in assisting pathologists in diagnosing blood disorders.

## Introduction

The morphological examination of bone marrow (BM) nucleated cells plays a crucial role in the diagnosis of various hematological diseases, including acute leukemia (AL), chronic leukemia (CL), myelodysplastic syndrome (MDS), plasma cell myeloma (PCM), and hemorrhagic disease. It is considered one of the most critical diagnostic procedures, alongside immunological diagnosis and cytogenetics diagnosis according to the diagnostic guidelines of hematopoietic cancers issued by the World Health Organization (WHO)^1–6^. Typically, BM morphology assessment involves skilled technicians performing a differential count followed by verification and diagnosis by experienced hematopathologists. However, this process heavily relies on the expertise of technicians and pathologists and is time-consuming, which limits the overall efficiency of BM assessment^7–9^. Therefore, there is a pressing need for an automated approach to conducting standardized BM cell differential counts.

A convolution neural network (CNN) is a kind of feedforward neural network that consists of convolution computation and depth structure^10^. CNNs, being representative algorithms of deep learning, have gained widespread usage in computer-aided systems^11^. Their exceptional ability to extract image features was showcased when CNNs achieved top performance in the ImageNet 2012 competition. Since then, numerous studies have concentrated on CNN development and its application in clinical settings, to reduce the workload of medical professionals in object recognition and image classification and improve the precision of clinical diagnosis^8,12–14^. Automated pathology analysis systems developed based on CNN models are mainly used for histopathology and peripheral blood morphology, and there are fewer reports on the identification of BM nucleated cells until the last five years when they have been rapidly developed ^14–17^. By combining CNN and Gabor, Huang et al. created the innovative MGCNN framework for classifying blood cells. In comparison to conventional CNNs, this unique approach significantly increases classification accuracy but comes at a higher computational cost^18^. Liu et al. were able to analyze 200 fields in 16 min using a faster Region-Convolutional Neural Network (R-CNN) for BM imaging cell detection, taking an average of 4.8 s per image and achieving an accuracy of 0.496. However, the microscope's focus had to be manually adjusted throughout the observation to maintain a clear field of view^19^. Eckardt et al. used a multi-step deep learning methodology to separate cells from pictures of BM to discriminate between acute myeloid leukemia (AML) and healthy cells and to forecast the state of the *Nucleophosmin 1* (*NPM1*) mutation, the most prevalent mutation in AML. However, this system requires the manual selection of areas for disease classification as judged by the pathologist, making the results potentially erroneous^20^. To detect acute lymphoblastic leukemia (ALL) in microscopic blood pictures, Atteia et al. are optimized using the Bayesian optimization technique. On a holdout test set, the best CNN model determined by the Bayesian optimization approach for ALL detection recorded 100% accuracy, specificity, and sensitivity^21^.

The Morphogo system we have developed overcomes many of these limitations, enabling efficient and accurate identification and classification of BM nucleated cells. According to our previous research, The Morphogo system integrates digital imaging of BM smear with artificial intelligence-based automatic BM cell differential count and has shown high accuracies in identifying various cell types, including granulocytic cells, erythroid cells, lymphoid cells, plasma cells, and monocytic cells, and even metastatic cancer cells^6,8,22,23^. We are committed to further improving the Morphogo system to enhance its performance and clinical value in assisting with the diagnosis of hematologic diseases.

## Methods

### Sources and classification of samples

This was a retrospective study. 508 BM cases were collected from Kingmed Diagnostics from October 2021 to December 2021. Following the recommendations of pathologists, the BM smears were divided into five groups, denoted G1–G5, based on the extent of pathological and cell morphological changes. The diseases grouped within each category are as follows: G1: Relatively normal cases; G2: Disorders with quantitative abnormalities primarily affecting mature cells, including anemia, bleeding/thrombosis, myeloproliferative neoplasms (MPN), chronic myeloid leukemia (CML); G3: Disorder follow-up cases; G4: Malignant hematological disorders characterized by a substantial proliferation of blasts and immature cells, including acute leukemia (AL), Multiple myeloma (MM); G5: Disorders associated with a higher occurrence of abnormal cells, including megaloblastic anemia (MgA), myelodysplastic syndrome (MDS), Chronic lymphoproliferative disease (CLPD). All BM smears underwent appropriate staining using the Wright-Giemsa method, ensuring the quality aligned with the recommendation of the nation guide to clinical laboratory procedures (NGCLP, fourth edition) or the international council for Standardization in Hematology (ISH)^8^. The study was approved by the Ethics Committee of Guangzhou Kingland Medical Laboratory Center. The detailed information of the enrolled BM cases was listed in Table 1.

**Table 1:** Grouping and basic information of BM smear samples.

Group	Sample	Number
G1	Relatively Normal	111
G2	Anemia	33
	Bleeding/Thrombosis	26
	MPN	11
	CML	11
	Others	17
G3	Disorder Follow-up	152
G4	AL	60
	MM	39
	Others	1
G5	MgA	6
	MDS	18
	CLPD	12
	Others	11

*MPN* myeloproliferative neoplasms, *CML* chronic myeloid leukemia, *AL* acute leukemia, *MM* multiple myeloma, *MgA* megaloblastic anemia, *MDS* myelodysplastic syndrome, *CLPD* chronic lymphoproliferative disease.

### System workflow

Morphogo system is a CNN-based Artificial Intelligence (AI) system developed by Hangzhou Zhiwei Information and Technology Ltd that is used to perform a differential count of BM nucleated cells automatically.

**Figure 1:**
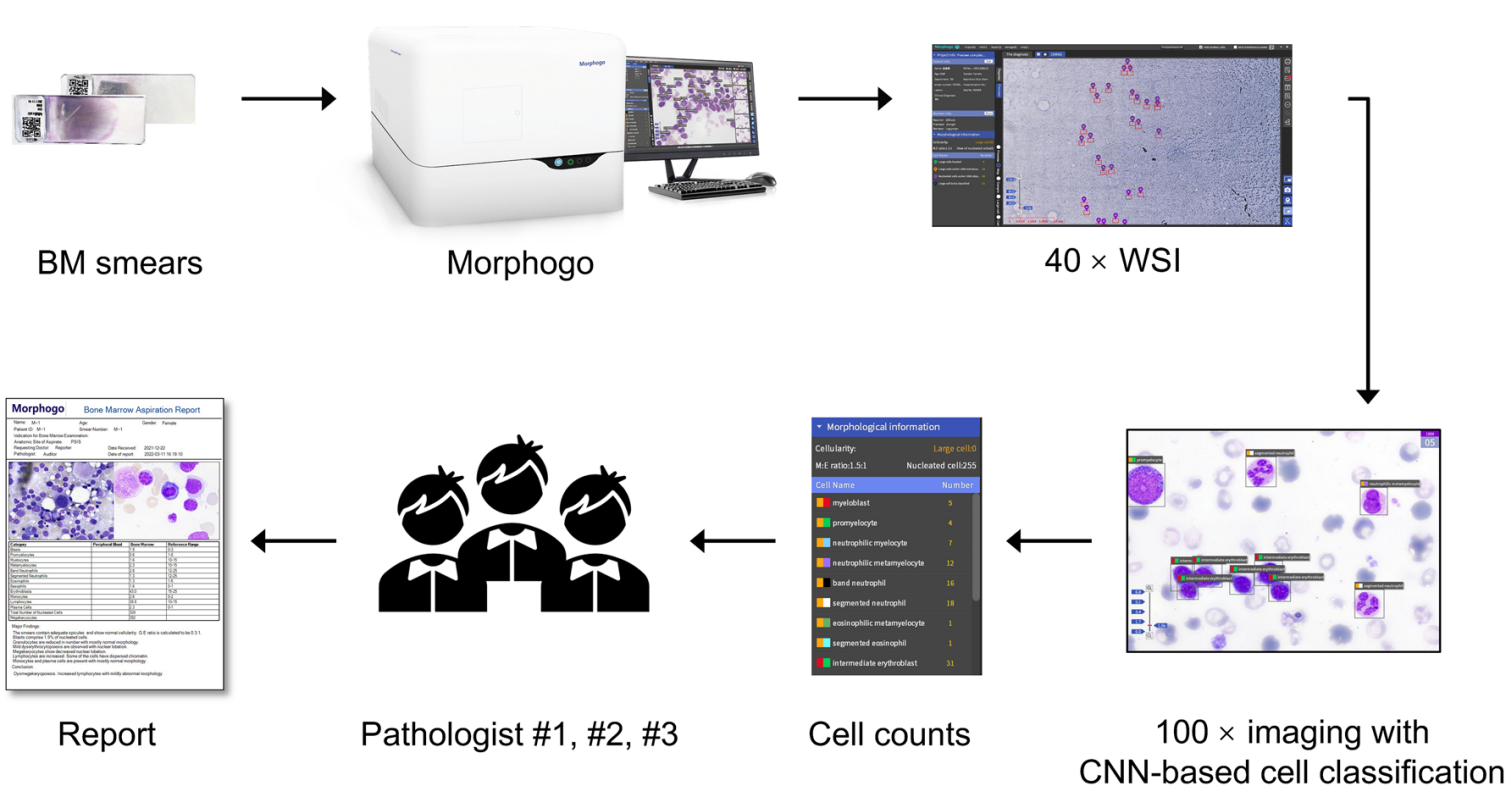
Workflow design for Morphogo analysis and pathologist review.

The workflow is as follows: (1) The Morphogo system initiates an automated scan of the BM smear using a 40 × objective lens, capturing a whole slide image (WSI) in the process. This enables the system to count megakaryocytes and identify the adaptive area for cell analysis. (2) Subsequently, the system switches to a 100 × objective lens to capture images of the designated area. Using CNN, the system identifies BM nucleated cells within this area and performs a differential cell count until a specified number of cells are obtained. (3) Before finalizing and releasing the cell morphology report, the data undergoes review by a pathologist. (Fig. 1).

### Morphogo system evaluation

**Figure 2:**
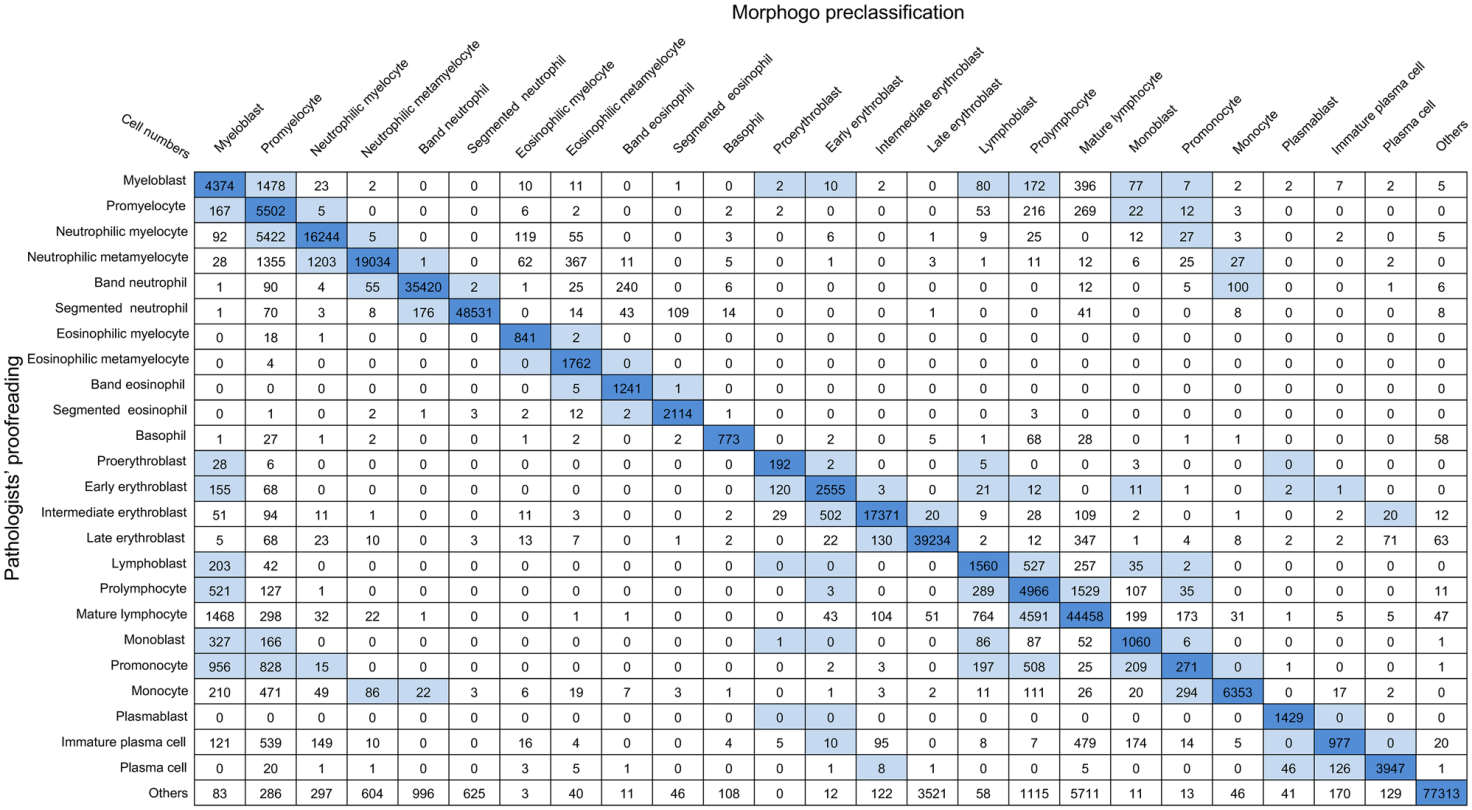
The summary of cell classification results obtained by Morphogo pre-classification and pathologists’ proofreading. The confusion matrix displays the count of cell images within each of the 25 morphological categories of BM nucleated cells. Rows represent the preliminary classifications by the Morphogo system, while columns reflect the pathologists' review. Diagonal entries in the matrix indicate the instances where the Morphogo system's classification aligns with the pathologists' review. Mix-ups that are considered tolerable are highlighted in light blue.

To evaluate the cell classification performance of the Morphogo system in different hematological diseases, the BM nucleated cells were categorized into 25 categories: proerythroblast, early erythroblast, intermediate erythroblast, late erythroblast, myeloblast, promyelocyte, neutrophilic myelocyte, neutrophilic metamyelocyte, band neutrophil, segmented neutrophil, eosinophilic myelocyte, eosinophilic metamyelocyte, band eosinophil, segmented eosinophil, basophil, monoblast, promonocyte, monocyte, lymphoblast, prolymphocyte, mature lymphocyte, plasmablast, immature plasma, plasma cell and others including smudge cell, histocyte, and mast cell according to WHO classification. Cell classification performance was evaluated in terms of sensitivity, specificity, positive predictive value (PPV), negative predictive value (NPV), and accuracy^14,24^. Accurately identifying individual morphological categories can be challenging, particularly when closely related categories exhibit morphological similarities. Recognizing this uncertainty in the morphological identification of BM nucleated cells, we incorporate the concept of tolerance classes, wherein certain mispredictions by the CNN model are deemed acceptable even if they differ from the precise labels provided by pathologists. This consideration was called tolerance classes^25^. The presence of tolerance classes is illustrated in Fig. 2, where the light blue color indicates tolerable mix-ups. For example, the confusion between myelocyte and promonocyte falls within the realm of tolerance. furthermore, we collected and compared the results of pathologists’ proofreading of all BM smears with the output of the Morphogo system, using kappa values as a metric to assess the agreement between the two approaches in disease diagnosis.

### Establishment of algorithms

In the process of Morphogo scanning and analyzing BM smears, intelligent algorithms play an important role. There are several key algorithms involved in this process. The first is the slide scanning area algorithm, which extracts the slide area to be observed by mimicking the human task-based visual object attention mechanism to determine the 40 × scanning coverage. The second is the auto-focal plane algorithm. When the camera rapidly captures more than 100 images with varying sharpness at different object distances, the Sobel operator is applied to extract the gradient values in different directions of the images. By quantifying image clarity using a dedicated function, the algorithm identifies the clearest regions within each image and an image fusion algorithm is then employed to merge these regions, ensuring the best clarity for every nucleated cell within the field of view.

Then, the 40 × full-slide assembling algorithm is utilized to introduce feature changes while maintaining a consistent scale, and the key points of the image are identified by the Gaussian differential function, and the key points are matched based on Ransac algorithm, achieving seamless assembly of the image and generating a WSI. Once the WSI is obtained, an area selection algorithm is used to select an optimal area for 100 × cell imaging. In 100 × cell images, a cell segmentation method based on saturation clustering is employed to accurately separate and locate the nucleated cells for differential count. Finally, the classification of BM nucleated cells is realized by a deep learning algorithm. This algorithm utilizes expert-labeled cell images and incorporates different types of cell morphological characteristics. By leveraging and the updated big data platform, which provides a continually expanding dataset, the algorithm achieves accurate classification and analysis of BM nucleated cells.

### Training of algorithm

The Morphogo system, which has been trained by more than 2.8 million BM nucleated cells, has now developed and refined to the point where it can automatically scan and analyze BM smears in less than 10 min while detecting more than 35 different types of nucleated cells (Table 2). The training of the algorithm was run on a server equipped with Intel Core i9 10, 900X, 16G × 4 ADATA DDR4, NVIDIA GeForce RTX 2080 Ti cards, and CUDA Version 10.2. The optimal algorithm for cell categorization was obtained after several training sessions. Subsequently, 385,207 BM cell images in this paper were used as validation datasets.

**Table 2:** Classes of BM cells pre-classified by Morphogo.

Number	Class of Cells
1	Myeloblast
2	Promyelocyte
3	Neutrophilic myelocyte
4	Neutrophilic metamyelocyte
5	Band neutrophil
6	Segmented neutrophil
7	Eosinophilic myelocyte
8	Eosinophilic metamyelocyte
9	Band eosinophil
10	Segmented eosinophil
11	Basophil
12	Proerythroblast
13	Early erythroblast
14	Intermediate erythroblast
15	Late erythroblast
16	Megaloblastic early erythroblast
17	Megaloblastic intermediate erythroblast
18	Megaloblastic late erythroblast
19	Lymphoblast
20	Prolymphocyte
21	Mature lymphocyte
22	Atypical lymphocyte
23	Monoblast
24	Promonocyte
25	Monocyte
26	Plasmablast
27	Immature plasma cell
28	Plasma cell
29	Histocyte
30	Smudge cell
31	Phagocyte
32	Mast cell
33	Erythrocyte
34	Mitosis
35	Platelet

### Statistical analysis and interpretation

Excel version 2016 was used to analyze the sensitivity, specificity, PPV, NPV, and accuracy of Morphogo’s cell classification by assuming pathologists’ annotations as the absolute true cell classification. The correlations of cell proportions were plotted by GraphPad Prism 7.0. Kappa and ICC for two different methods were performed by IBM SPSS Statistics 20 to evaluate the consistency. To interpret the correlation, the r-value is as follows: r less than 0.09 was no correlation; 0.1–0.3 was a weak correlation; 0.3–0.5 a was a moderate correlation. 0.5–1.0 was a high correlation^26^. The relationship between K value and consistency is as follows: K = 0–0.20, extremely weak consistent; K = 0.21–0.40, weak consistent; K = 0.41–0.60, moderately consistent; K = 0.61–0.80, strongly consistent, and K = 0.81–1.0, almost perfect consistent^27^. Unless otherwise indicated, all data were displayed as mean and standard deviation (x̅ ± s) and analyzed by two-tailed Student’s t-test. *p* < 0.05 were considered statistically significant differences.

### Statement

All of the above methods were performed by the relevant guidelines and regulations.

### Ethical approval

This study was approved by the Ethics Committee of Guangzhou Kingmed Diagnostics Medical Laboratory Center. Because abandoned samples of routine clinical detections were collected and clinical case information was used, the Ethics Committee of Guangzhou Kingmed Diagnostics Medical Laboratory Center therefore has approved the application for performing the study with the exemption of informed consent from all participants.

## Results

### Highly accurate classification of BM nucleated cells by Morphogo system

**Figure 3:**
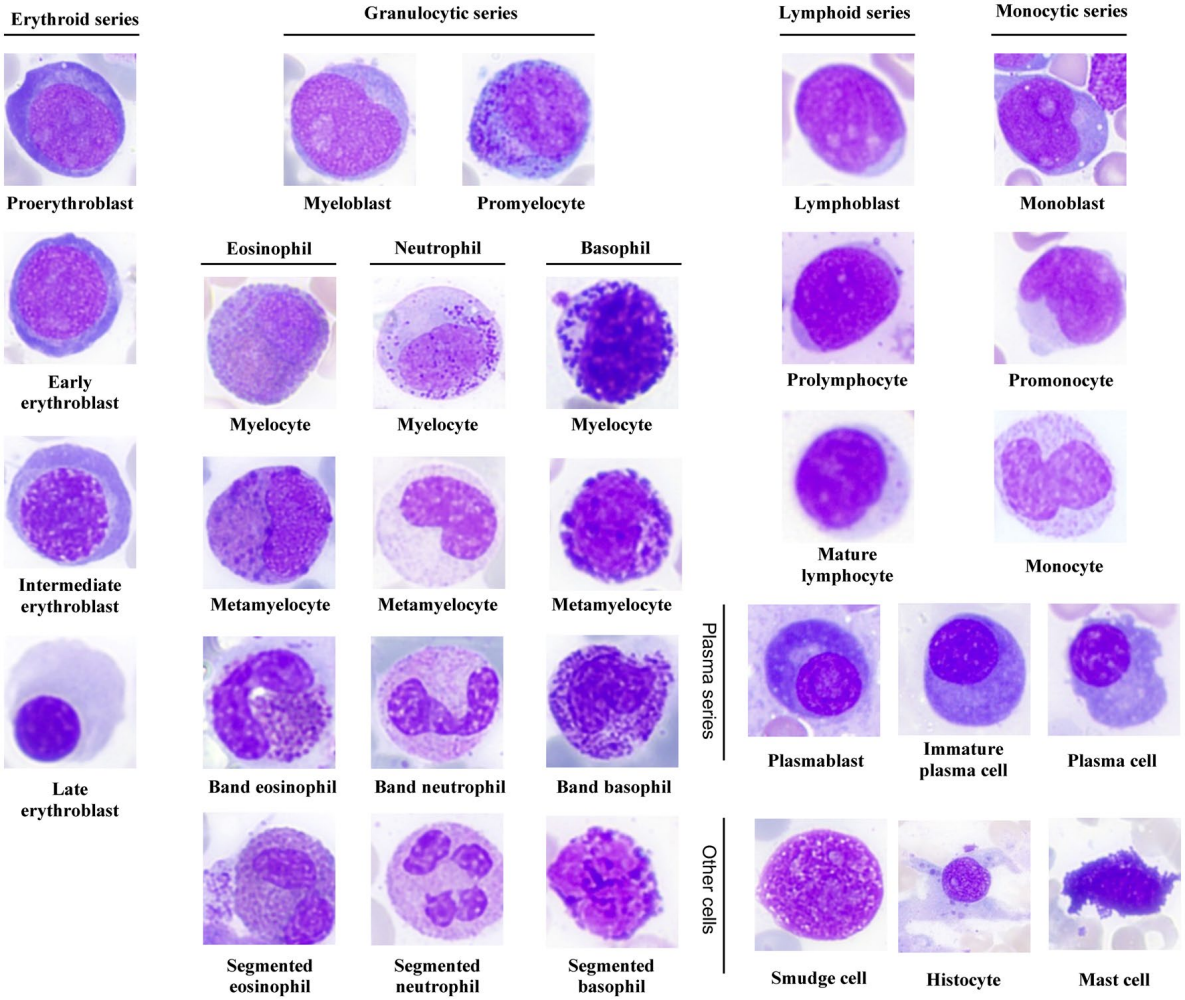
Sample images of BM cells classified by Morphogo.

The high-resolution digital images of BM nucleated cells from the ROI were acquired using the Morphogo system. These cell images were categorized into 25 categories (Fig. 3). Cell classification results predicted by the Morphogo system and annotated by pathologists were shown in a confusion matrix (Fig. 2). The dataset consisted of 385,207 single-cell images. The row displayed cell classification results from the Morphogo system, and the column showed results from pathologists’ proofreading. The dark blue pane located diagonally illustrated the number of nucleated cells classified by the Morphogo system which were entirely consistent with pathologists’ proofreading. The white pane represented cells that were classified as different types by the Morphogo system and pathologists proofreading. Cell numbers shown in light blue panes represented cells that were easily confused either between different maturing stages within the same lineage or between morphologically related cell types, so their misclassification was considered tolerable.

To evaluate the cell classification performance of the Morphogo system under different pathological conditions, the Morphogo system was applied to patient cases with more than 14 types of hematological diseases. The evaluation indicators were calculated for each disease condition and are shown in Table 3. The sensitivity in the classification of BM nucleated cells by the Morphogo system was an average value of 80.95%. The Morphogo system exhibited a sensitivity of more than 95% in the identification of 9 categories of BM nucleated cells. For specificity, the test sample yielded an average of 99.48% for all classes of BM nucleated cells. The value of PPV varied greatly in different classes of BM nucleated cells, ranging from 30.45% to 99.69%, with an average value of 76.49%. The Morphogo system showed a more than 95% PPV value among Neutrophilic metamyelocytes, Band neutrophils, Segmented neutrophils, Intermediate erythroblasts, Monocytes, and others. The average value of the NPV was more than 99%, ranging from 95.43 to 100.00%. And the NPVs of eosinophilic metamyelocyte, band eosinophil, and plasmablast ahead of the other cells have a value of 100.00%. The Morphogo system performed a high accuracy in the classification of BM nucleated cells by 95.55–99.98%, with an average value of 99.01%. Therefore, the results of our study showed that the Morphogo system had high sensitivity, specificity, PPV, NPV, and accuracy in the classification and counting of BM nucleated cells.

**Table 3:** Performance of Morphogo system to classify BM nucleated cells.

Class of Cells	Number of Cells	Sensitivity (%)	Specificity (%)	PPV (%)	NPV (%)	Accuracy (%)
Myeloblast	6663	65.65	98.83	49.75	99.39	98.26
Promyelocyte	6261	87.88	96.97	32.40	99.79	96.82
Neutrophilic myelocyte	22,030	73.74	99.50	89.93	98.42	98.03
Neutrophilic metamyelocyte	22,154	85.92	99.78	95.93	99.15	98.98
Band neutrophil	35,968	98.48	99.66	96.73	99.84	99.55
Segmented neutrophil	49,027	98.99	99.81	98.71	99.85	99.71
Eosinophilic myelocyte	862	97.56	99.93	76.87	99.99	99.93
Eosinophilic metamyelocyte	1766	99.77	99.85	75.43	100.00	99.85
Band eosinophil	1247	99.52	99.92	79.70	100.00	99.92
Segmented eosinophil	2141	98.74	99.96	92.84	99.99	99.95
Basophil	973	79.45	99.96	83.93	99.95	99.91
Proerythroblast	236	81.36	99.96	54.70	99.99	99.95
Early erythroblast	2949	86.64	99.84	80.55	99.90	99.74
Intermediate erythroblast	18,278	95.04	99.87	97.37	99.75	99.64
Late erythroblast	40,030	98.01	98.96	91.58	99.77	98.86
Lymphoblast	2626	59.41	99.58	49.46	99.72	99.31
Prolymphocyte	7589	65.44	98.02	39.86	99.30	97.37
Mature lymphocyte	52,295	85.01	97.21	82.70	97.64	95.55
Monoblast	1786	59.35	99.77	54.39	99.81	99.58
Promonocyte	3016	8.99	99.84	30.45	99.29	99.13
Monocyte	7717	82.32	99.94	96.43	99.64	99.58
Plasmablast	1429	100.00	99.98	93.77	100.00	99.98
Immature plasma	2637	37.05	99.91	74.64	99.57	99.48
Plasma cell	4166	94.74	99.94	94.45	99.94	99.88
Others	91,361	84.62	99.92	99.69	95.43	96.29

Sensitivity = TP/(TP + FN) * 100%; Specificity = TN/(TN + FP) * 100%; PPV = TP/(TP + FP) * 100%.

NPV = TN/(FN + TN) * 100%; Accuracy = (TP + TN)/(TP + FP + TN + FN) *100%.

*TP* true positive, *TN* true negative, *FP* false positive, *FN* false negative.

### Morphogo system was in substantial agreement with pathologists’ proofreading in the identification of BM nucleated cells

**Figure 4:**
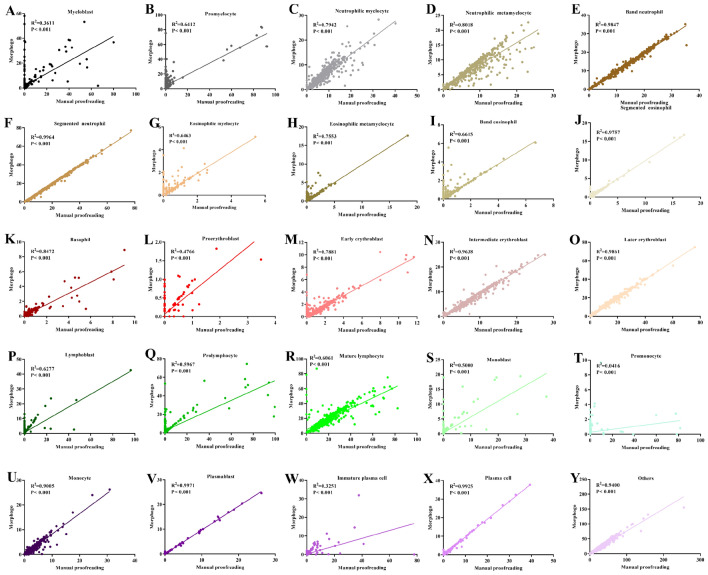
The correlation analysis of Morphogo pre-classification and pathologists’ proofreading. (**A**)–(**Y**) shows the scatter plot of linear regression lines of the percentage of BM cells after paired counting of BM smears in 508 patients.

To better understand the agreement of BM nucleated cells between the Morphogo system and pathologists proofreading, we performed the correlation analysis and consistency analysis between the Morphogo system and pathologists proofreading in the classification and counting of BM nucleated cells. The results were shown in Fig. 4**.** Morphogo system showed positive correlation between pathologists and Morphogo system in the classification of myeloblast (r = 0.6009, Fig. 4A), promyelocyte (r = 0.8008, Fig. 4B), neutrophilic myelocyte (r = 0.8912, Fig. 4C), neutrophilic metamyelocyte (r = 0.8954, Fig. 4D), band neutrophil (r = 0.9923, Fig. 4E), segmented neutrophil (r = 0.9982, Fig. 4F), eosinophilic myelocyte (r = 0.8039, Fig. 4G), eosinophilic metamyelocyte (r = 0.8691, Fig. 4H), band eosinophil (r = 0.8134, Fig. 4I), segmented eosinophil (r = 0.9878, Fig. 4J), basophil (r = 0.9204, Fig. 4K), proerythroblast (r = 0.6903, Fig. 4L), early erythroblast (r = 0.8878, Fig. 4M), intermediate erythroblast (r = 0.9817, Fig. 4N), late erythroblast (r = 0.9930, Fig. 4O), lymphoblast (r = 0.7923, Fig. 4P), prolymphocyte (r = 0.7724, Fig. 4Q), mature lymphocyte (r = 0.7785, Fig. 4R), monoblast (r = 0.7071, Fig. 4S), promonocyte (r = 0.2038, Fig. 4T), monocyte (r = 0.9489, Fig. 4U), plasmablast (r = 0.9985, Fig. 4V), immature plasma cell (r = 0.5702, Fig. 4W), plasma cell (r = 0.9963, Fig. 4X) and others (r = 0.9695, Fig. 4Y), the *P* values of these 25 classes of BM nucleated cells were less than 0.001.

It was shown that the cell classification results of the Morphogo system were in general agreement with that of pathologists proofreading in the identification of BM nucleated cells, as evidenced by kappa value (0.461–0.987), except for promonocytes (Table 4). The Morphogo system exhibited almost perfect agreement with pathologists’ proofreading in the classification of neutrophilic metamyelocyte, band neutrophil, segmented neutrophil, eosinophilic myelocyte, eosinophilic metamyelocyte, band eosinophil, segmented eosinophil, basophil, early erythroblast, intermediate erythroblast, late erythroblast, mature lymphocyte, monocyte, plasmablast, plasma cell, and others, with the kappa value of more than 0.813. However, the classification of myeloblast, promyelocyte, lymphoblast, prolymphocyte, monoblast, and immature plasma showed only moderate agreement between the Morphogo system and pathologists’ proofreading, with Kappa value from 0.461 to 0.566. Overall, correlation and consistency results collectively supported that the Morphogo system maintained a substantial agreement with pathologists’ proofreading in identifying BM nucleated cells.

**Table 4:** Evaluation of the consistency between Morphogo pre-classification and pathologists’ proofreading of BM cells using the Cohen kappa coefficient.

Class of Cells	Kappa	*P* value
Myeloblast	0.557	0.000
Promyelocyte	0.461	0.000
Neutrophilic myelocyte	0.800	0.000
Neutrophilic metamyelocyte	0.901	0.000
Band neutrophil	0.973	0.000
Segmented neutrophil	0.987	0.000
Eosinophilic myelocyte	0.860	0.000
Eosinophilic metamyelocyte	0.858	0.000
Band eosinophil	0.885	0.000
Segmented eosinophil	0.957	0.000
Basophil	0.816	0.000
Proerythroblast	0.654	0.000
Early erythroblast	0.834	0.000
Intermediate erythroblast	0.960	0.000
Late erythroblast	0.940	0.000
Lymphoblast	0.536	0.000
Prolymphocyte	0.483	0.000
Mature lymphocyte	0.813	0.000
Monoblast	0.566	0.000
Promonocyte	0.136	0.000
Monocyte	0.886	0.000
Plasmablast	0.968	0.000
Immature plasma	0.493	0.000
Plasma cell	0.945	0.000
Others	0.892	0.000

### The Morphogo system has high application value in the diagnosis of hematological diseases

To further verify the application value of the Morphogo system in the diagnosis of hematological diseases, the diagnoses made based on the Morphogo system were compared to the pathologists proofreading. The evaluation was made for each sample group (G1–G5) in terms of intraclass correlation coefficient (ICC) and 95% CI. As shown in Table 5, except for the progenitors, ICC between the two different methods was high for granulocytes, erythrocytes, lymphocytes, monocytes, and plasma cells in the G1, G2, G3, and G5 groups (ICC ≥ 0.818, *P* < 0.01), and slightly lower for G4. Based on these results, the diagnosis results of the Morphogo system for most hematological diseases should be correct.

**Table 5:** Correlation analysis between Morphogo and manual proofreading among 5 groups (ICC and 95% CI).

Cell Series	G1	G2	G3	G4	G5
Blasts	0.083 (− 0.104–0.265)	0.593 (0.992–0.996)	0.216 (0.060–0.363)	0.835 (0.764–0.886)	0.203 (− 0.086–0.461)
Granulocytes	0.995 (0.992–0.996)	0.998 (0.997–0.998)	0.995 (0.994–0.997)	0.855 (0.791–0.900)	0.996 (0.992–0.998)
Erythroblasts	0.996 (0.994–0.997)	0.989 (0.984–0.993)	0.996 (0.995–0.997)	0.995 (0.993–0.997)	0.989 (0.980–0.994)
Lymphocytes	0.956 (0.937–0.970)	0.893 (0.844–0.927)	0.912 (0.880–0.935)	0.531 (0.374–0.658)	0.831 (0.715–0.902)
Monocytes	0.944 (0.919–0.961)	0.818 (0.740–0.874)	0.980 (0.972–0.985)	0.815 (0.737–0.871)	0.945 (0.903–0.969)
Plasma cells	0.943 (0.918–0.961)	0.992 (0.988–0.995)	0.998 (0.997–0.998)	0.826 (0.752–0.880)	0.896 (0.820–0.941)

*ICC* intraclass correlation coefficient.

### The Morphogo system automatically records the time it takes to scan BM smears and identify BM cells

Morphogo system can complete automatic scanning continuously, and efficiently, with a success rate of 99.4%. The average time of a single slide scan is 7:46 (min), and most of the slide scanning time is concentrated in 5–9 min. The Morphogo system takes 7.46 ± 0.002 min/sheet to identify and count BM cells (Table 6). These results suggest that the Morphogo system can assist in the artificial diagnosis of hematologic diseases, which greatly saves time.

**Table 6:** The time required for Morphogo to scan a digital BM slide and count 600 nucleated cells.

Method	Time (min)
Morphogo	7.46 ± 0.002

## Discussion

One of the most challenging steps in the workup of diagnosis of blood diseases is the morphological classification of BM nucleated cells, and the effectiveness of the classifier determines its utility in blood disorder diagnostics. CNN models, currently the leading classification framework, have shown superior performance compared to manual cellular morphological feature detection ^8,25,28^ in recognizing and classifying diverse medical images. Our results, obtained using Morphogo, a cell morphology analysis system created using CNN models, indicate that rapid advancements in artificial intelligence will enable automated hematologic disease screening systems to realize their full potential.

To enhance the CNN’s ability to discern potential relationships between BM nucleated cells during the learning process, we trained the CNN on the discriminative features of BM nucleated cells using 2.3 million BM cell images. We then tested the trained model on over 0.5 million cell images collected from various hospitals. This extensive database is beyond the reach of most models. The Morphogo system can now identify more than 35 classes of BM nucleated cells, including certain pathological cell types, and a few non-hematopoietic cells. Our results showed that the Morphogo system achieves high sensitivity, specificity, PPV, NPV, and accuracy in the classification and counting of 25 classes of BM nucleated cells. Moreover, the Morphogo system’s cell differential results were in substantial agreement with those of pathologists’ proofreading. Furthermore, the Morphogo system has the capability to automatically scan, identify and count BM nucleated cells, with an average processing time of 7.46 min. This indicates a substantial potential for the Morphogo system to enhance the efficiency of BM cell morphology analysis.

The study provided pathologists with a potential application of AI in the morphology examination of BM smears. However, as previous research has reported, even experienced pathologists find it challenging to identify small differences between cells with similar morphological characteristics are difficult to identify^25^. For example, a promonocyte is often misidentified as a monocyte. Both manual counting and smart counting are affected by staining differences, and increasing the training data does not significantly improve accuracy^29^. The current Morphogo system cannot accurately distinguish subtle differences between morphologically similar cells. This limitation may explain why the sensitivity and PPV performance were not satisfactory in the identification of promonocytes. Furthermore, the image quality of BM nucleated cells depends on several factors such as the quality of BM smear preparation, the pathological condition, and the imaging process^13^. These factors can contribute to inaccuracies in BM cell identification. The morphology of blasts in AL of G4 is more uniform, while in MDS of G5, blasts tend to be polymorphic and malformed^13^. Consequently, blasts are easier to be identified and classified in AL, and difficult to be identified in MDS, which might be the cause of the higher misdiagnosis rate in some cases when using the Morphogo system compared to pathologists’ manual review. However, due to the large number of BM samples processed daily and the laborious and time-consuming nature of BM cell differential counting, some laboratories only count 100–200 cells in each BM smear. By utilizing the Morphogo system, they can review AI-based cell differential count results on a computer screen, dramatically improving the efficiency of laboratory work. The Morphogo system can analyze a larger number of cells in a shorter time, allowing pathologists to review more cells and avoid misdiagnosing critical morphological changes, ultimately reducing the misdiagnosis rate. Furthermore, the Morphogo system provides a standardized and digital approach to cell differential counting, enabling more reliable and repeatable assessment of morphology, and enhancing the overall quality control of BM morphology assessment. It also facilitates better comparison and communication among technicians and pathologists, ultimately leading to more effective patient care.

This study employed a single-center method, where all BM smears were prepared in the same laboratory and digitally processed. The performance evaluation of the Morphogo system focused on identifying BM nucleated cells in common hemato-pathological conditions, and the dataset reasonably reflects the morphological changes of most cell types. However, this study still had some limitations. Firstly, it was limited to 14 common diseases, and the number of cases was insufficient to determine whether the Morphogo system’s AI performance would be consistent across all common hematopathological diseases and rare conditions. Secondly, efforts should be made to minimize the impact of staining variations on categorization strategies. Last but not least, we did not specifically collect samples of cells with dysplastic abnormalities during the initial development of the algorithm, serving as a training sample. As a result, when performing the statistical analysis, we discovered that the quantities of various types of qualitative cellular changes were not sufficient. In the future, further studies should be conducted using a larger number of BM samples that encompass a broader range of hematological diseases from multiple laboratories. This would help to further validate the BM cell identification performance of the Morphogo system more comprehensively.

## Conclusion

CNN-based Morphogo system could classify and count BM nucleated cells to assist pathologists to diagnose hematological diseases. The Morphogo system is a potential digital analysis system that provides a more objective and efficient method for BM morphology assessment.

### Supplementary Information

Supplementary Information.

## Data Availability

All data generated or analyzed during this study are included in this manuscript and supplementary information files.
